# A prospective, observational clinical trial on the impact of COVID-19-related national lockdown on thyroid hormone in young males

**DOI:** 10.1038/s41598-021-86670-9

**Published:** 2021-03-29

**Authors:** Giulia Brigante, Giorgia Spaggiari, Barbara Rossi, Antonio Granata, Manuela Simoni, Daniele Santi

**Affiliations:** 1 Unit of Endocrinology, Department of Biomedical, Metabolic and Neural Sciences, University of Modena and Reggio Emilia, via Campi 287, 41125 Modena, Italy; 2 Unit of Endocrinology, Department of Medical Specialties, Azienda Ospedaliero-Universitaria of Modena, Ospedale Civile of Baggiovara, via Giardini 1355, 41126 Modena, Italy

**Keywords:** Endocrinology, Signs and symptoms

## Abstract

Trying to manage the dramatic coronavirus disease 2019 (COVID-19) infection spread, many countries imposed national lockdown, radically changing the routinely life of humans worldwide. We hypothesized that both the pandemic per se and the consequent socio-psychological sequelae could constitute stressors for Italian population, potentially affecting the endocrine system. This study was designed to describe the effect of lockdown-related stress on the hypothalamic-pituitary-thyroid (HPT) axis in a cohort of young men. A prospective, observational clinical trial was carried out, including patients attending the male infertility outpatient clinic before and after the national lockdown for COVID-19 pandemic. The study provided a baseline visit performed before and a follow-up visit after the lockdown in 2020. During the follow-up visit, hormonal measurements, lifestyle habits and work management were recorded. Thirty-one male subjects were enrolled (mean age: 31.6 ± 6.0 years). TSH significantly decreased after lockdown (p = 0.015), whereas no significant changes were observed in the testosterone, luteinising hormone, follicle-stimulating hormone, estradiol and prolactin serum levels. No patient showed TSH serum levels above or below reference ranges, neither before nor after lockdown. Interestingly, TSH variation after lockdown was dependent on the working habit change during lockdown (p = 0.042). We described for the first time a TSH reduction after a stressful event in a prospective way, evaluating the HPT axis in the same population, before and after the national lockdown. This result reinforces the possible interconnection between psychological consequences of a stressful event and the endocrine regulation.

## Introduction

The coronavirus disease 2019 (COVID-19) pandemic represents the most serious health and social emergency in recent decades. At the beginning of 2020, most European countries decided to fight against the exponential spread of the infection closing non-essential recreational and productive activities, enforcing a national lockdown. In Italy, the lockdown was imposed by decree of the President of the Council of Ministers on March 8th, until the gradual reduction of the restrictive measures starting from May 4th. Thus, many citizens have experienced a radical change in their lifestyle habits for about two months. This phenomenon led to short and long term psychosocial and mental health implications^1,2^. Indeed, the initial acute stress due to the fear of contagion has been superimposed on a subsequent chronic stress secondary to the limitation of freedom of movement and social life, with a complex revolution of the daily routine^3^. Not surprisingly, the individual psychological health resulted largely affected during the national lockdown, with a documented increase in severe anxiety syndrome incidence^4^.

It is well established that the human body activates several processes to cope psychological and/or physical stressful events, in which endocrine system plays a crucial role^5^. Although the hypothalamic–pituitary–adrenal axis remains the main actor in the endocrine regulation of stress response^6^, stress-induced alteration of the hypothalamic-pituitary-thyroid (HPT) axis has been demonstrated both in animals and in humans. In animal models, a decrease in serum thyroid stimulating hormone (TSH) levels was observed in response to both acute and chronic intermittent stress, while the daily rhythm of TSH secretion resulted not impaired^7,8^. On the other hand, active thyroid hormones triiodothyronine (T3) and tetraiodothyronine (T4) resulted significantly lower consequently to repeated exposure to stress^9^. A similar HPT axis activation pattern was highlighted in humans presenting the so-called “non-thyroidal illness syndrome”^10^. In case of acute physical stress, as observed in a large proportion of hospitalized patients, decreased serum levels of free T3 (fT3) and free T4 (fT4) accompanied by inappropriately low/normal TSH levels were recorded, as a metabolic defence mechanism^11^. However, HPT axis modifications in response to psychological stress are even more obscure, and scantly investigated in humans. Several authors detected a T3 elevation and a modest TSH reduction in post-traumatic stress disorders in women with childhood sexual abuse^12^, in combat veterans^13^, and in female breast cancer patients^14^. Similar results were recently confirmed in subjects who experienced an earthquake swarm, one year after the event, with a significant reduction in TSH levels, even more evident for the epicentre-nearest population^15^.

With this in mind, we consider the COVID-19-related lockdown as a potential stressful event for young Italian men. In particular, this study aims to describe for the first time the effect of lockdown-related stress on the HPT axis in a cohort of young male subjects.

## Materials and methods

**Figure 1:**
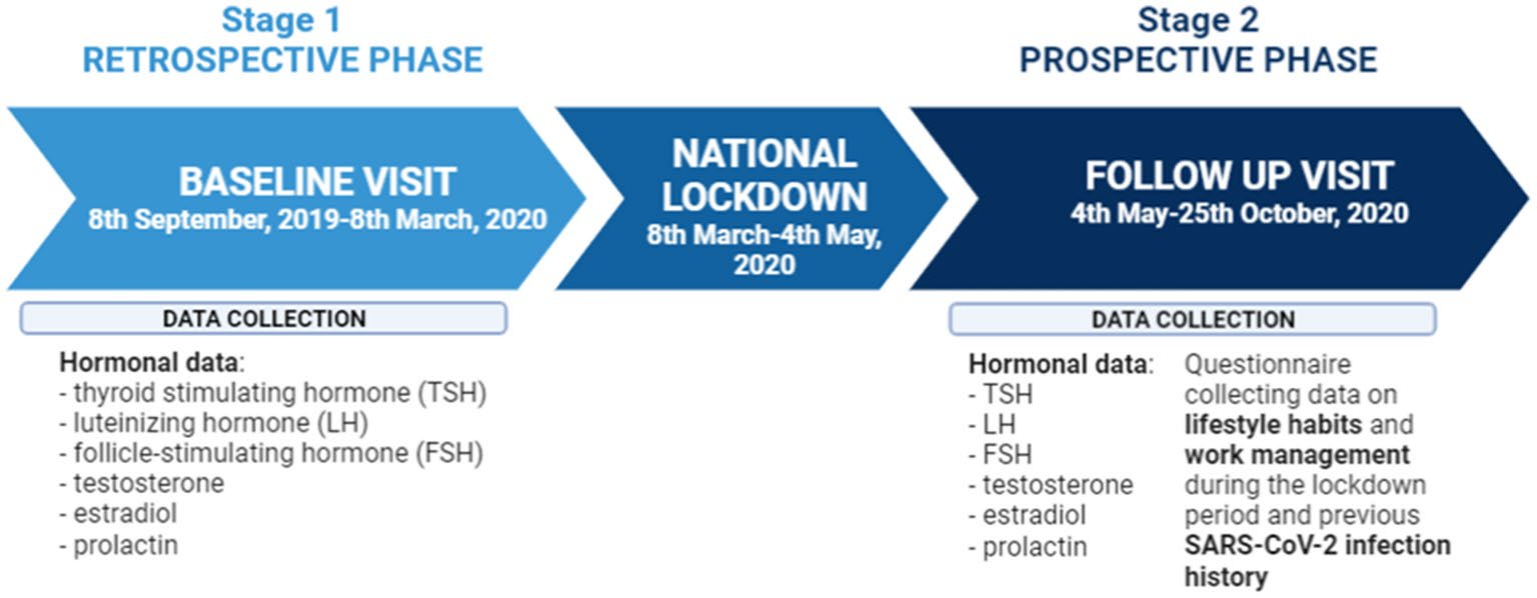
Flow-chart of the study. SARS-CoV-2 = severe acute respiratory syndrome coronavirus-2.

A prospective, observational clinical trial was carried out at the Unit of Endocrinology of the Department of Biomedical, Metabolic and Neural Sciences of the University of Modena and Reggio Emilia (Modena, Italy). All patients attending the male infertility outpatient clinic before and after the first national lockdown for COVID-19 pandemic (lasting from March 8th, 2020 to May 4th, 2020) were considered eligible. The enrolment started at the end of the first lockdown when nationally-imposed restrictions on non-urgent medical activity were removed (May 4th, 2020) (Fig. 1). Originally, the study design provided the evaluation of patients every four months after the lockdown restrictions removal. However, the dramatic national situation due to the severe acute respiratory syndrome coronavirus-2 (SARS-CoV-2) spread increase forced the Italian government to impose a new quarantine the following Autumn (October 25th, 2020). Thus, we decided to close prematurely the study to avoid any possible interference of a second national lockdown on hormonal homeostasis, thus collecting only a single post-lockdown evaluation (Fig. 1). In the first phase of the study, potentially eligible patients have been selected among outpatient reports available on the Hospital Information System. All patients who attended the male infertility outpatient clinic in the six months before March 8th, 2020, i.e. the quarantine start, were evaluated for inclusion and exclusion criteria (Fig. 1). Eligible patients were males over 18 years of age with a TSH measurement performed at least once in the six months before the COVID-19 lockdown. Exclusion criteria were: (i) previous or current treatment for hypo- or hyperthyroidism, (ii) thyroid surgery history, (iii) previous metabolic radiotherapy with radioactive Iodine (Iodine-131), (iv) concomitant or previous pituitary pathology (i.e. hypopituitarism, pituitary adenoma, chromosomal or genetic disorders potentially affecting pituitary function) and (v) other serious systemic comorbidities (i.e. renal failure, hepatic failure, active or previous oncological disease). Hence, the study design provided a retrospective baseline visit performed within the six months before the lockdown, while the prospective follow-up visit was performed between two and four months after the lockdown end (May 4th, 2020). From the retrospective baseline visit, hormonal data about thyroid function, pituitary–gonadal axis and prolactin levels were extracted, since these parameters are routinely assessed in the work-up of male infertility. During the prospective follow-up visit, next to hormonal values, additional data regarding lifestyle habits and work management during the lockdown period were recorded by a questionnaire, as well as previous SARS-CoV-2 infection history. This questionnaire (supplementary material) did not provide a specific score, but it was used only to collect information about the pre- and post-lockdown patients’ habits.

During the two visits, the following hormonal parameters were recorded: TSH, testosterone, estradiol, luteinizing hormone (LH), follicle-stimulating hormone (FSH) and prolactin. FT3 and fT4 serum levels were available only in post-lockdown visit, since these hormones were not routinely measured in the infertility management in case of TSH levels within the reference ranges. Thus, these parameters were not considered in the comparison between pre- and post-lockdown visits.

### Ethical statement

Authors declare that all procedures were in accordance with the ethical standards of the Helsinki Declaration of 1975 as revised in 2013. The study protocol was approved by the Ethics Committee of “Area Vasta Emilia Nord” (protocol number: AOU0019719/20 of 15/07/2020), and all participating subjects provided their written informed consent for the enrolment.

### Laboratory methods

TSH serum levels were measured by chemiluminescent microparticle immunoassay (Abbott Diagnostics, USA), with intra-assay coefficient of variation (CV) of 3.10% and an inter-assay CV of 3.50%. FT3 serum levels were measured by chemiluminescent microparticle immunoassay (Abbott Diagnostics, USA), with intra-assay CV of 2.80% and an inter-assay CV of 3.65%. FT4 serum levels were measured by chemiluminescent microparticle immunoassay (Abbott Diagnostics, USA), with intra-assay CV of 3.80% and an inter-assay CV of 5.70%.

Total testosterone serum levels were measured by Chemiluminescent Microparticle Immunoassay (Architect, Abbott, Dundee, UK), with inter- and intra-assay coefficients of variation (CV) of 5.2 and 5.1%, respectively. FSH and LH were measured by Chemiluminescent Microparticle Immunoassay (Architect, Abbott, Longford, Ireland) with inter- and intra-assay CV of 4.1 and 3.1% for LH, and 4.6 and 4.2% for FSH, respectively. Serum estradiol were measured by Chemiluminescent Microparticle Immunoassay on the ARCHITECT platform (Abbott Laboratories), with a sensitivity of 0.6 pg/mL. PRL was measured by Chemiluminescent Immunoassay (Beckman Coulter, Brea, CA, USA) with inter- and intra-assay CV of 4.2% and 1.6%, respectively.

### Statistical analysis

Data distribution was evaluated by Kolmogorov–Smirnov test. Continuous data were compared before and after lockdown using ANOVA univariate analysis for normally distributed parameters and Mann–Whitney U-test for not-normally distributed parameters.

Multivariate linear regression analyses were performed considering TSH serum levels after lockdown as dependent variable and patients’ age, body mass index (BMI), time elapsed after quarantine, weight change and other hormones available as independent variables. In order to identify those parameters able to predict the TSH variation after lockdown, the number of patients with a TSH decrease was calculated. This categorical variable was used as dependent variable in logistic regression analyses, using as co-variables patients age, BMI, time elapsed after quarantine and weight change after lockdown, while changes in lifestyle habits (such as smoke, alcohol and eating behaviour) were considered as cofactors. Moreover, since we recently demonstrated that TSH serum levels show an annual seasonality^16^, we adjusted logistic regression analyses, considering the season in which the TSH measurement was performed.

Statistical analyses were performed using the ‘Statistical Package for the Social Sciences’ (SPSS) software for Windows (version 25.0; SPSS Inc, Chicago, IL). For comparisons, p < 0.05 was considered statistically significant.

## Results

Thirty-one male subjects were enrolled, with a mean age of 31.6 ± 6.0 years. As reported above, all patients have been evaluated for couple infertility, 22 (71%) for primary and 9 (29%) for secondary infertility. Seven patients (22.6%) showed comorbidities, such as hypertension (2 patients, 6.5%), dyslipidaemia (2 patients, 6.5%), gastritis (2 patients, 6.5%), and minor depression (1 patient, 3.1%). Anamnestic characteristics are summarized in Table 1.

**Table 1:** Anamnestic characteristics of the 31 subjects enrolled in the study.

	Baseline characteristics (n = 31)
**Smoking habit**	
Actual smokers *n (%)*	8 (25.8)
Number of smoked cigarettes *(mean* ± *standard deviation)*	15.7 ± 9.7
Former smokers *n (%)*	8 (25.8)
**Alcohol consumption**	
Habitual alcohol drinkers *n (%)*	26 (83.9)
Alcohol units daily drunk *(mean* ± *standard deviation)*	0.94 ± 0.5
**Regular habitual physical activity** *n (%)*	12 (38.7)

The retrospective pre-lockdown evaluation was performed a mean of 5.6 ± 3.9 months before the restriction measures beginning, whereas the post-lockdown a mean of 3.5 ± 1.4 months after the blockage removal. In the post-lockdown, a significant TSH reduction was detected in 26 patients (83.9%) (p = 0.015), whereas no significant changes were observed in the other evaluated hormones (Table 2). The post-hoc analysis of the mean TSH difference highlighted a statistical power of 71%. No patient showed TSH serum levels above or below reference ranges, neither before nor after lockdown. Similarly, fT3 and fT4 serum levels were within normal ranges in all patients enrolled at the follow-up visit (3.7 ± 1.7 and 9.7 ± 1.8, respectively). Moreover, no patients showed testosterone serum levels below normal range (3.0 ng/mL) or prolactin higher than reference ranges (17 ng/mL) neither at baseline, nor after lockdown.

**Table 2:** Hormonal differences between pre- and post-lockdown evaluations. Data are expressed as mean ± standard deviation. Bold characters represent significantly different variables.

	Reference range	Pre-lockdown	Post-lockdown	p-value
TSH (microIU/mL)	0.35–4.94	2.3 ± 1.2	1.7 ± 0.7	**0.015**
Testosterone (ng/dL)	2.2–7.8	5.1 ± 1.6	5.1 ± 1.7	0.928
LH (IU/L)	1–9	3.9 ± 3.7	4.1 ± 2.6	0.863
FSH (IU/L)	1–12	6.3 ± 8.9	5.8 ± 3.7	0.796
Estradiol (pg/mL)	11–44	18.5 ± 10.3	19.1 ± 13.0	0.843
Prolactin (ng/mL)	3–13	13.2 ± 11.3	11.2 ± 8.9	0.447

FSH = follicle-stimulating hormone; LH = luteinizing hormone; TSH = thyroid stimulating hormone. The statistical comparison was performed by Mann–Whitney U-test.

Interestingly, no documented cases of SARS-CoV-2 infection were reported in our group of patients. Although only 5 patients (16.1%) were subjected to the nasopharyngeal swab and 9 (29%) to serological evaluation, all these diagnostic procedures resulted negative for SARS-CoV-2 infection. Only one patient (3.2%) reported a positive case in his family. During the lockdown, 14 subjects (45.2%) worked from home, whereas usual work habits changed in 6 more subjects (19.4%). Comprehensively, 20 subjects (64.5%) had to radically change their working habits during the national restriction. This period significantly impacted also the habitual lifestyle. Indeed, 9 subjects (29%) reduced their usual physical activity, 4 (12.9%) increased the number of smoked cigarettes, 5 (16.1%) increased the daily alcohol intake, and 21 (67.7%) changed their habitual eating habits. Interestingly, we recorded a significant weight increase of 1.9 ± 3.2 kg during the lockdown.

Multivariate analyses showed that TSH serum levels after lockdown were not dependent on patients’ age, BMI, time elapsed after lockdown, weight change and hormones measured (fT3, fT4, testosterone, LH, FSH, estradiol and prolactin) (p = 0.675). However, logistic regression analyses highlighted that the working habit change during lockdown significantly predict the TSH decrease after lockdown (p = 0.042) (Table 3). This result is extremely interesting, suggesting that the TSH decrease is evident mainly in those patients that had to change their work habit. These results remained significant also after adjustment for the season in which TSH was measured.

**Table 3:** Logistic regression analyses performed considering the TSH change after national lockdown as dependent variable. Bold characters represent statistically significant results.

	B	Standard error	p-value	Exp(B)	95% confidence interval	
					Lower limit	Upper limit
Intercept	33.54	0.000	–	–	–	–
Weight change	17.04	1.47	**0.042**	3.017	1.023	5.062
Patient’s age	− 4.05	36.90	0.999	–	–	–
Free T3	− 7.49	62.57	0.999	–	–	–
Free T4	2.79	24.88	0.999	–	–	–
Testosterone	− 2.34	21.15	0.999	–	–	–
Luteinizing hormone (LH)	6.44	32.38	0.999	–	–	–
Follicle-stimulating hormone (FSH)	5.32	29.57	0.999	–	–	–
Estradiol	3.12	15.63	0.999	–	–	–
Prolactin	− 2.12	11.32	0.999	–	–	–
Body mass index	6.87	56.55	0.999	–	–	–
Time elapsed after quarantine	8.12	63.59	0.999	–	–	–
Diet habit change	− 3.59	28.97	0.999	–	–	–
Physical activity change	− 1.73	9.86	0.999	–	–	–
Alcohol consumption change	2.56	21.68	0.999	–	–	–
Smoking habit change	3.49	25.41	0.999	–	–	–

Finally, correlation analyses showed no significant correlation between continuous variables collected and TSH serum levels before and after the lockdown, suggesting that the TSH change is mainly due to other factors, such as stress.

## Discussion

This observational prospective study demonstrates a significant decrease in TSH serum levels in young males after the restrictions and life changes imposed by the national lockdown to contain the COVID-19 pandemic in Italy. In particular, we highlight a TSH decrease of 0.6 microIU/mL after two months of lockdown, suggesting that the HPT axis is highly sensitive to stressful events in humans. We could speculate that the HPT axis activation is mediated by the sympathetic and the adrenal “fight-or-flight” system to physiologically face prolonged life-threatening situations^17^. We recently demonstrated that an acute stressful event, such as the earthquake swarm in northern of Italy, significantly reduced TSH serum levels^15^. However, all previous studies evaluated different population before and after the stressful event^18–20^. It is extremely difficult, indeed, to perform a prospective clinical trial that allow the evaluation of hormonal changes pre- and post-stressor in the same enrolled subjects. Here, instead, we were able, for the first time, to evaluate the same young men before and after a clearly stressful event, such as the COVID-19-related lockdown. No studies so far performed a prospective rigorous evaluation of TSH change after the national lockdown imposed for SARS-CoV-2 pandemic.

Previous available studies in humans detected a TSH decrease accompanied by a peripheral thyroid hormones increase in post-traumatic stress disorders^14,21–24^. In our trial, all subjects, even if in follow up for a condition of endocrine interest, such as couple infertility, presented normal thyroid function before lockdown, as well as prolactin serum levels that is a known factor affecting the HPT axis. However, although the TSH reduction after lockdown is statistically significant, it does not lead to values below the reference range or to symptoms clinically attributable to disthyroidism. Moreover, TSH alteration is not accompanied by an increase in peripheral thyroid hormone levels. This is not surprising given that TSH changes are known to precede change in thyroid hormones, since the pituitary receptor of the latter is very sensitive and therefore able to induce feedback mechanisms before thyroid hormone levels go beyond the reference range^25^. Indeed, in post-lockdown visit, the fT3 and fT4 serum levels remain within reference ranges. When the HPT axis is intact, fT4 and TSH are related by an inverse log-linear relationship: small changes in fT4 result in large changes in serum TSH concentrations^26^. Thus, serum TSH levels are considerably more sensitive than direct thyroid hormone measurements for assessing HPT axis alteration. On the one hand the lowering of TSH could anticipate and amplify an initial alteration of thyroid gland function, on the other hand it could also suggest a primary reduction of TSH secretion, as hypothesized in the non-thyroidal illness syndrome.

The mechanism by which the experience of lockdown leads to a reduction in TSH is not easy to understand. Our analyses demonstrate that this hormonal variation does not depend on age, time elapsed since the end of the restrictions or the change in body weight. Furthermore, the result remains statistically significant even after adjusting for the season in which the blood collection was performed. Indeed, we previously suggested that TSH has a semi-annual seasonal pattern with higher levels in summer and winter^16^. The factor that mainly predict the lowering of TSH in our cohort is the change in work habits, which was experienced by a high percentage of the examined subjects. Most of them had to radically change their daily routine, working from home, with little or no physical contact with colleagues, exploiting new technologies and adapting their homes to workplaces. Moreover, several studies recently confirmed the potential stressful capability of SARS-CoV-2-related national lockdown in several clinical contexts^27–32^. Our hypothesis is that these changes, combined with the general context of the pandemic and the reduction of social contacts, has led to some psychological stress affecting the HPT axis. Obviously, this is not a direct measure of the stress, but it could be considered an indirect marker since there are no reliable markers capable of quantifying a stressful event.

Since the baseline data were collected retrospectively, a direct measurement of stress levels before and after lockdown was precluded, but we can reasonably assume that changing daily routines and new job challenges caused some degree of stress in our patients. Regarding work stress, data on the effect of difficulties resulting from the reshaping of work during the COVID-19 lockdown are not available yet. With the necessary differences, an interesting starting point could be given by the literature on the effects of burnout on the HPT axis. However, unfortunately, even the results in this field are scarce and inconclusive^33^. Moreover, literature data suggest a possible role of acute stress on the onset and course of some autoimmune diseases, including thyroid diseases, such as Basedow-Graves hyperthyroidism^34^. However, Basedow-Graves’ disease has a clinically explosive onset and it is characterized by an increase in thyroid hormones, as well as a lowering of TSH. We therefore ruled out this possibility in all subjects enrolled in this study.

Our results are limited by the small sample size, due to a resumption of the COVID-19 pandemic in Italy with further new restrictions. Moreover, the relevance of our result is limited by the lack of a control group, which is unavoidable for this topic because the whole population was subjected to the same restrictions and those who were not (i.e. health workers) were subjected to stress anyway, albeit in a different way. Moreover, we are not able to really measure the stress lived by each enrolled man. In particular, we do not used psychometric tools to objectify the individual stress, since no questionnaires have been validated so far for this kind of pandemic situation. However, we could speculate that the national lockdown is a stressful event that affect all young men enrolled in a comparable way. Moreover, men of couple seeking fertility could be prone to chronic stress because of the difficulty to have child. However, the stress related to infertility has not been demonstrated in male partner of infertile couples so far. And if there is, it could not be a bias for the study, since the same condition (i.e. infertility) has been maintained in both visits considered. Future studies should focus to directly quantify the real stress weight on human well-being. Again, we cannot rule out that the TSH change discovered in our study is due to anti-thyroid antibodies or pre-existing thyroid nodular goitre, which could impair thyroid function and consequently TSH. However, the potential presence of anti-thyroid antibodies and of nodules should not have changed between the two visits considered in the study, which were temporally close to each other. Moreover, a potential stress-related activation of thyroiditis or nodules would have led to a significant change in hormonal values, up to the onset of overt hyperthyroidism which, however, we do not find in our series. Thus, future studies are needed to confirm the autoimmune activation after stressful event. Finally, many other factors could affect hormonal homeostasis, both measurable and not. However, we enrolled a homogeneous casuistry, including young men without severe comorbidities, evaluated before and after a single stressful event. Thus, possible confounding factors are reasonably affecting enrolled subjects both before and after lockdown. One important confounding factor should be COVID-19 infection itself. Previous studies demonstrated a possible hypothalamic-pituitary involvement in SARS leading to central hypothyroidism and/or central hypocortisolism^35^. Even very recent studies on SARS-CoV-2 show a significant decrease in serum TSH levels in affected patients compared to healthy controls and non-COVID-19 pneumonia patients^36,37^. However, these data were referred to hospitalized subjects and none of the young men enrolled in the present study had a story of admission for SARS-CoV-2.

In conclusion, we evaluated for the first time the TSH change after a stressful event in a prospective way, evaluating the HPT axis pre- and post- national lockdown in the same young men. Our results demonstrate a significant decrease in TSH serum levels mainly in those patients that had to change their work habit, suggesting that- lockdown restrictions can have not only physiological but also organic consequences on endocrine axes functionality.

## Supplementary Information

Supplementary Information
