# Supplementary material for: A prospective, observational clinical trial on the impact of COVID-19-related national lockdown on thyroid hormone in young males
Source: Sci Rep. 2021 Mar 29;11:7075. doi: 10.1038/s41598-021-86670-9 (PMC8007755; doi:10.1038/s41598-021-86670-9)
Supplement: Supplementary file 1 — Supplementary Information [file 41598_2021_86670_MOESM1_ESM.doc]

**Supplementary material**

Questionnaire used to collect information pre- and post-national lockdown, created to evaluate the psychological impact of the SARS-CoV-2-related lockdown. The questionnaire was reported as used, in Italian language.

**DATI ANAGRAFICI**

Nome:

Cognome:

Data di nascita:

Data della visita:

**DATI RELATIVI ALL’INFEZIONE**

Ha eseguito il tampone oro-faringeo per SARS-CoV-2? Sì NO se sì, con quale esito:

□ positivo

□ negativo

Ha eseguito lo screening sierologico per SARS-CoV-2? Sì NO se sì, con quale esito:

□ positività IgM

□ positività IgG

□ positività IgM e IgG

□ negativo

In famiglia ha avuto casi di infezione da SARS-CoV-2? Sì NO

Durante il periodo di isolamento sociale ha avuto sintomi simil-influenzali? Sì NO

In famiglia ha avuto casi di familiari con sintomi simil-influenzali? Sì NO

**INQUADRAMENTO PROFESSIONALE**

Attuale professione:

Durante il periodo di lockdown ha continuato a lavorare come in precedenza? Sì NO

Se Sì, ci sono state modifiche o riduzioni dell’orario lavorativo? Sì NO se sì, quali:

Se No, per quanto tempo non ha lavorato?

Se No, da quando ha ripreso a lavorare?

Con quante persone vive attualmente?

Con quante perone viveva prima del lockdown?

Con quante persone ha vissuto il periodo di isolamento sociale?

**INQUADRAMENTO FARMACOLOGICO**

Assumeva farmaci prima dell’isolamento sociale? Sì NO se sì, quali:

Ha assunto nuovi farmaci durante l’isolamento sociale? Sì NO se sì, quali:

Attualmente quali farmaci assume?

**STILI DI VITA**

Pratica regolarmente sport e/o attività fisica? Sì NO se sì, quale:

Durante l’isolamento sociale ha continuato a praticare attività fisica? Sì NO

se sì, con quale frequenza:

□ quotidiana

□ settimanale

□ occasionale

Fuma? Sì NO se sì, quante sigarette al giorno:

Durante l’isolamento sociale ha modificato la sua abitudine tabagica? Sì NO se sì, come:

□ aumentata

□ ridotta

Beve alcolici? Sì NO se sì, con quale frequenza

□ quotidiana

□ settimanale

□ occasionale

Durante l’isolamento sociale ha modificato la frequenza con cui consuma alcolici? Sì NO se sì, come:

□ aumentata

□ ridotta

Il suo peso corporeo attuale?

Il suo peso prima del lockdown?

Durante il lockdown ha modificato le sue abitudini alimentari? Sì No se sì, come:

□ ho mangiato in maniera meno corretta

□ ho mangiato in maniera più corretta

□ ho mangiato di più

□ ho mangiato di meno
